# Spatially Covarying Patterns of Gray Matter Volume and Concentration Highlight Distinct Regions in Schizophrenia

**DOI:** 10.3389/fnins.2021.708387

**Published:** 2021-10-14

**Authors:** Kelly Rootes-Murdy, Elaheh Zendehrouh, Vince D. Calhoun, Jessica A. Turner

**Affiliations:** ^1^Department of Psychology, Georgia State University, Atlanta, GA, United States; ^2^Neuroscience Institute, Georgia State University, Atlanta, GA, United States; ^3^Department of Computer Science, Georgia State University, Atlanta, GA, United States; ^4^Tri-Institutional Center for Translational Research in Neuroimaging and Data Science (TReNDS), Georgia State University, Georgia Institute of Technology, Emory University, Atlanta, GA, United States

**Keywords:** schizophrenia, multivariate analysis, Jacobian scale modulation, gray matter concentration (GMC), gray matter volume (GMV)

## Abstract

**Introduction:** Individuals with schizophrenia have consistent gray matter reduction throughout the cortex when compared to healthy individuals. However, the reduction patterns vary based on the quantity (concentration or volume) utilized by study. The objective of this study was to identify commonalities between gray matter concentration and gray matter volume effects in schizophrenia.

**Methods:** We performed both univariate and multivariate analyses of case/control effects on 145 gray matter images from 66 participants with schizophrenia and 79 healthy controls, and processed to compare the concentration and volume estimates.

**Results:** Diagnosis effects in the univariate analysis showed similar areas of volume and concentration reductions in the insula, occipitotemporal gyrus, temporopolar area, and fusiform gyrus. In the multivariate analysis, healthy controls had greater gray matter volume and concentration additionally in the superior temporal gyrus, prefrontal cortex, cerebellum, calcarine, and thalamus. In the univariate analyses there was moderate overlap between gray matter concentration and volume across the entire cortex (*r* = 0.56, *p* = 0.02). The multivariate analyses revealed only low overlap across most brain patterns, with the largest correlation (*r* = 0.37) found in the cerebellum and vermis.

**Conclusions:** Individuals with schizophrenia showed reduced gray matter volume and concentration in previously identified areas of the prefrontal cortex, cerebellum, and thalamus. However, there were only moderate correlations across the cortex when examining the different gray matter quantities. Although these two quantities are related, concentration and volume do not show identical results, and therefore, should not be used interchangeably in the literature.

## Introduction

Schizophrenia is a severe mental illness characterized by cognitive, behavioral, and emotional dysfunction (American Psychiatric Association, 2013). Numerous structural MRI studies report cortical reductions in individuals with schizophrenia compared to healthy controls in the frontal and temporal lobes, thalamus, and cerebellum (McCarley et al., 1999; Wright et al., 2000; Shenton et al., 2001; Honea et al., 2005; Glahn et al., 2008; Segall et al., 2009; Turner et al., 2012; Gupta et al., 2015). The current understanding is that the reductions are largely clustered in certain regions and not uniform throughout the brain (McCarley et al., 1999; Shenton et al., 2001; Kubicki et al., 2002), though more subtle effects can be found with larger samples (van Erp et al., 2013, 2018). With sufficient sample size (N ∼ 10,000) the entire cortex shows reduced thickness in schizophrenia (van Erp et al., 2018). However, in individual studies the results can largely depend on pre-processing protocols, statistical analyses, and sample sizes (Senjem et al., 2005; Silver et al., 2011; Chen et al., 2014; Radua et al., 2014). Study-specific decisions in pre-processing protocols can result in different smoothing kernel sizes, segmentation parameters, and the addition of Jacobian-scaled modulation. All of these decisions can ultimately change the resulting estimates of diagnostic group differences (Eckert et al., 2006; Radua et al., 2014).

### Voxel Based Morphometry

Voxel based morphometry (VBM) involves segmentation of structural MRI images and spatial normalization of these images to the same stereotactic space, usually for voxel-wise analysis of gray matter values (Ashburner and Friston, 2000). There are two estimates of gray matter used in VBM studies of gray matter differences in schizophrenia, that we consider for the purposes of this paper: concentration and volume. Concentration can be thought of as the proportion of gray matter to other tissue types within a given voxel, whereas volume is the absolute amount of gray matter in each voxel, as originally described in Ashburner and Friston, 2000. The quantification of gray matter, either concentration or volume, is determined by the addition of Jacobian-scaled modulation (as described below) during the pre-processing step of segmentation. This step of modulation allows for the preservation of gray matter volume by multiplying the images by the relative voxel volumes or the Jacobian determinants of the deformation field, explained in more detail in the previous literature (Ashburner and Friston, 2000; Radua et al., 2014).

### Pre-processing Without Modulation

Following spatial normalization, the global differences in brain shape are removed (Eckert et al., 2006; Bora et al., 2011), and the resulting images are interpreted as gray matter concentration (Ashburner and Friston, 2000). These values represent the probability that the voxels in question ascribe as gray matter (Good et al., 2001). Gray matter concentration is not the comparison of relative concentration of gray or white matter structures in the spatially normalized images, but rather the proportion of gray matter assigned to those tissue types; it can also be useful for identifying cortical regions with poor registration (Mechelli et al., 2005).

### Pre-processing With Jacobian-Scaled Modulation

Pre-processing involves spatial normalization that contracts or expands brain regions to match a pre-identified template (Ashburner and Friston, 2000). Jacobian-scaled modulation accounts for the amount of contraction needed to move voxels in the native space image to corresponding voxels in the template by multiply of the spatially normalized gray matter by its relative volume before and after spatial normalization: I_b_ = I_a_ × (V_a_/V_b_) (V_a_ = volume before normalization, V_b_ = volume of the template (Jacobian determinants of deformation field), I_a_ = spatially normalized intensity, and I_b_ = intensity in signal after modulation) (Mechelli et al., 2005). In other words, the Jacobian determinants are the measures of warping applied to each image to match the template. The modulation step preserves local volumes of gray matter that were detected prior to normalization. For example, if a brain region is enlarged to match the standard template during normalization, then the partial volumes should be proportionally reduced to maintain the original amount of volume (Radua et al., 2014).

The use of Jacobian-scaled modulation is consistently cited in the literature examining degenerative diseases or atrophy, as it can ensure that inter-subject alignment preserves the intergroup differences (Eckert et al., 2006). However, there are some drawbacks to consider when including Jacobian-scaled modulation. Modulation can decrease the sensitivity to detect mesoscopic abnormalities due to the cluster-based statistics, as well as introduce multiplicative noise (Radua et al., 2014). Modulation also results in increased variance across groups when compared to non-modulation (Eckert et al., 2006; Meda et al., 2008; Segall et al., 2009; Radua et al., 2014; Torres et al., 2016). On the other hand, Jacobian-scaled modulation may help to analyze macroscopic regional differences in the absolute volume of gray matter as these differences might be removed during the non-linear registration but are re-introduced with the modulation (Good et al., 2001; Keller et al., 2004; Ranlund et al., 2018). The relationship between regional differences is important for understanding not only diagnostic differences but also potential medication effects or developmental trajectories (Gupta et al., 2015).

### Source Based Morphometry

Source-based morphometry (SBM) is a multivariate extension of VBM (Xu et al., 2009). SBM identifies patterns which covary among the participants whereas the VBM approach identifies gray matter differences through a voxelwise comparison. In SBM, each image is decomposed into a linear combination of components through an independent component analysis (ICA) and then normalized by multiplying the weight of each of those components in each participant’s image (Xu et al., 2009). Simply, it is a linear model with the sum of component maps and participant loadings making up the input segmentation maps. In that sense there is a direct link between the SBM information and the input voxel information (either concentration or volume). This technique results in components or patterns of gray matter variation for all subjects included in the analysis, which can be analyzed for differences among the groups in question. The contribution of component for each participant, or the individual loading coefficient, indicates that that pattern of gray matter variation is more strongly weighted for that individual (Xu et al., 2009). SBM can be applied to both concentration and volumetric gray matter images. For the purposes of the current study, we utilized SBM analyses in addition to the univariate VBM analyses.

### Study Overview

There are inconsistencies in the current schizophrenia literature on the use of Jacobian modulation in the pre-processing pipeline of MRI images. In addition, a number of studies do not cite either step in the methods section or use concentration and volume interchangeably, thus creating an unclear picture of what the results are depicting (Gur et al., 1999; Kubicki et al., 2002; Brent et al., 2016). Given the steps of both pre-processing pipelines, there is an assumption that the resulting images do not differ significantly and therefore, both classifications of gray matter (concentration or volume) should yield similar regional conclusions. The main difference between the Jacobian-scaled modulation and non-modulation is that modulated images represent the regional differences in the absolute amount, or volume, of gray matter and the unmodulated images represent the regional differences in the concentration of gray matter as it reported per unit volume in native space (Radua et al., 2014).

For the purposes of this study, the modulated images will be referred to as gray matter volume and unmodulated images will be referred to as gray matter concentration. We sought to examine the differences between gray matter concentration and volume within a schizophrenia dataset, and present how modifying the signal intensity of a voxel (to account for the expansion/contraction during the process of standardizing into Montreal Neurological Institute (MNI) coordinate space) can result in differences in gray matter findings.

## Materials and Methods

### Participants

Imaging and behavioral data were from the Center for Biomedical Research Excellence (COBRE) dataset, accessed *via* COINS and Schizconnect [RRID:SCR_010482,^1^^,2^; (Scott et al., 2011)]. Details of the participant recruitment can be found in Aine et al. (2017). The variables included in analyses were diagnosis of schizophrenia, determined by the Structural Clinical Interview for DSM-4; smoking status, determined from self-report responses on the Fagerstrom Test for Nicotine Dependence (FTND) (Heatherton et al., 1991); the mean translational motion corrections estimated from the resting state scan from the same dataset; intracranial volume (ICV) determined from Freesurfer segmentations (Fischl, 2012); olanzapine equivalents for current medication (Aine et al., 2017); gender; and age. Three participants were removed prior to analyses because their images did not meet quality assurance standards (correlation *r* < 0.90 when compared to the MNI template). Seven participants were removed for missing responses on smoking status (*N* = 2) or for missing resting state images (*N* = 5) because of head motion correction. Motion during functional scans is stable within individuals, negatively correlated to quality of structural scans, and can produce a potential confound on structural MRI measurements (Savalia et al., 2017) and therefore, resting state images were used for head motion correction. Following data curation, sixty-six adults with schizophrenia (54 males; mean age 38.08 ± 14.21 years) and 79 healthy control adults (59 males; mean age 37.66 ± 11.79 years) were included in the present study. See Table 1 for more participant demographic information.

**TABLE 1:** Participant demographic information.

	SZ (*N* = 65)	HC (*N* = 79)	Total (*N* = 145)	*p*
Males (%)	53 (81.81%)	62 (78.48%)	116 (80%)	0.30
Smokers (%)	23 (34.85%)	14 (17.72%)	37 (25.52%)	0.02*
Age ± SD	38.08 ± 14.21	37.66 ± 11.79	37.85 ± 12.90	0.85
Head motion ± SD	0.40 ± 0.28	0.46 ± 0.37	0.43 ± 0.33	0.33
OLZ ± SD	15.47 ± 11.11	–	–	–

SD, Standard Deviation; HC, healthy controls; SZ, individuals with schizophrenia; OLZ, total olanzapine equivalent dose; *p < 0.05.

#### MRI Acquisition and Preprocessing

High resolution T_1_-weighted images were acquired on a Siemens 3T TIM Trio scanner with a five-echo multi-echo MPRAGE (MEMPR) sequence [TE = 1.64, 3.5, 5.36, 7.22, 9.08 ms, TR = 2.53 s, inversion time = 1.2 s, 7° flip angle, number of excitations (NEX) = 1, slice thickness = 1 mm, FOV = 256 mm, resolution = 256 × 256].

We utilized the optimized VBM pre-processing procedure and SPM12 for image pre-processing and statistical analysis (Good et al., 2001). Images were normalized to MNI space with DARTEL and segmented in SPM12, a high-dimensional normalization pipeline. The non-brain tissues were stripped and gray matter, white matter, and cerebral spinal fluid (CSF) were segmented and normalized. We completed the preprocessing pipeline in two parallel steps to create both the gray matter concentration and gray matter volume images. Following segmentation, gray matter volume images were then smoothed to an 8 mm × 8 mm × 8 mm Gaussian kernel. Both VBM and SBM analyses were performed with the gray matter concentration and volume images.

### Voxel Based Morphometry Analysis

We performed a univariate VBM analysis on the dataset using SPM12^3^. In two separate general linear models (GLMs) for smoothed gray matter concentration and volume images, we added the covariates of age, gender, smoking status, intracranial volume (ICV), and average head motion. A second linear regression model examined the significant voxelwise results for olanzapine equivalents effects in individuals with schizophrenia. Statistical results for group comparisons (healthy controls > schizophrenia) were thresholded at a *q* < 0.001 with family discovery rate (FDR) correction.

Following VBM preprocessing, we utilized a voxelwise correlation on all voxels that contained ≥ 20% gray matter and intersected between the two sets of images (concentration and volume), to identify the correlations between gray matter concentration and volume.

### Source-Based Morphometry Analysis

The preprocessed images went through component estimation using the minimum description length algorithm (Rissanen, 1978). For the concentration images, there were 14 distinct gray matter components and for the volume images, there were 12 distinct gray matter components computed by the infomax algorithm (Bell and Sejnowski, 1995). We used the SBM module of the GIFT Toolbox^4^ to perform independent component analysis (ICA) decompositions (Xu et al., 2009) on the dataset. ICASSO (Himberg et al., 2004) with 20 ICA runs was used to ensure the stability of components.

A multivariate analysis of covariance (MANCOVA) model using R v3.6.2 (R Core Team, 2016) was completed with SBM components’ loading coefficients as dependent variables, diagnosis as a factor, gender and smoking status as dummy-coded covariates, and age, intracranial volume (ICV), and average head motion as covariates in separate models. A secondary linear regression model examined the significant case/control spatial maps for olanzapine equivalents effects in individuals with schizophrenia. We utilized a threshold of *q* < 0.05 with false discovery rate (FDR) correction.

Following the SBM analysis, we performed Pearson’s correlations on the ICA concentration spatial patterns (14 total) against the volume spatial patterns (12 total). We also performed a Jaccard Index to determine similarity between the two thresholded (*z* ≥ | 2|) concentration and volume images using the spatial patterns. Briefly, Jaccard Index is a measurement of similarity between samples that is defined as the size of the intersection divided by the size of the union of those samples (Real and Vargas, 1996).

## Results

### Voxel Based Morphometry Results

#### Effect of Diagnosis

**FIGURE 1:**
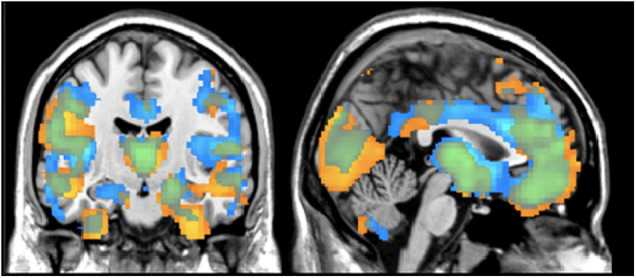
Regional Differences in Gray Matter Results from VBM analysis showing differences between healthy controls and individuals with schizophrenia in terms of gray matter concentration (blue) and gray matter volume (yellow). Results are thresholded at *z* > | 2| (*p* < 0.05, FDR corrected).

We evaluated relative voxelwise concentration and volume differences between diagnostic groups using a FDR correction. In Figure 1, the images show an overall pattern of gray matter reduction in individuals with schizophrenia. For the concentration results, the maximal differences were in the left insula (*p* < 0.001), the orbitofrontal cortex/Brodmann Area 11 (*p* < 0.001), and the right primary visual cortex (*p* < 0.001). For the volume images, the maximal differences were located in the temporopolar area (*p* < 0.001) and the left fusiform gyrus (*p* = 0.043). There was no significant effect of olanzapine equivalents in either the concentration or volume images. See Figure 1 and Table 2 for more details on group differences.

**TABLE 2:** Peak levels and MNI coordinates for significant voxel-based morphometry results.

Gray matter	Peak T	Peak *x, y, z*	Brain location	# Of voxels	p*
Concentration	7.35	−36, 18, −2	L Insula	6,193	1.53e-05
	7.19	−4, 44, −20	OFC/BA11	12,098	1.62e-05
	4.97	10, −62, 10	R Calcarine	848	0.0011
Volume	6.85	34, 22, −30	Temporopolar area/R BA38	3,088	1.32e-04
	5.23	−46, −60, −4	Left fusiform gyrus	389	0.0434

L, left; R, right; OFC, Orbitofrontal cortex; BA, Brodmann Area; *FDR corrected p < 0.05.

#### Effect of Quantity

**FIGURE 2:**
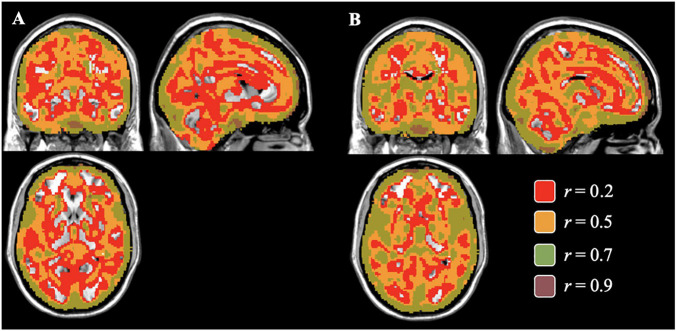
Voxelwise Correlations between Concentration and Volume for Controls **(A)** and Individuals with Schizophrenia **(B)** Colored correlations set at different thresholds between gray matter concentration and volume images of healthy controls **(A)** and individuals with schizophrenia **(B)** in the brain. The areas of overlap between the concentration and volume results were found mostly in areas of gray matter and gradually decreasing in the subcortical regions as the correlation threshold increases.

The average voxelwise correlation per individual between the gray matter concentration and volume images was *r* = 0.56, largely seen in voxels containing mostly gray matter. We separately compared the concentration and volume images by diagnosis to examine the pattern differences between diagnostic groups. Images from the healthy controls had an average correlation of *r* = 0.51, *p* < 0.05 (max *r* = 0.99) and images from the schizophrenia group had an average correlation of *r* = 0.60, *p* = 0.03 (max *r* = 0.97). To visualize the distribution of correlation, different levels of correlations were mapped onto corresponding voxels. Figure 2 shows those voxelwise correlations between concentration and volume in healthy controls (A) and individuals with schizophrenia (B). Negative correlations in both maps made up less than 4% of the total number of voxels and therefore, were not included in the visual mapping. As the correlation threshold was increased from *r* = 0.2 to *r* = 0.9, overlap between the two sets of images dropped to the outer edges of the cortex.

### Source-Based Morphometry Results

#### Effect of Diagnosis

**FIGURE 3:**
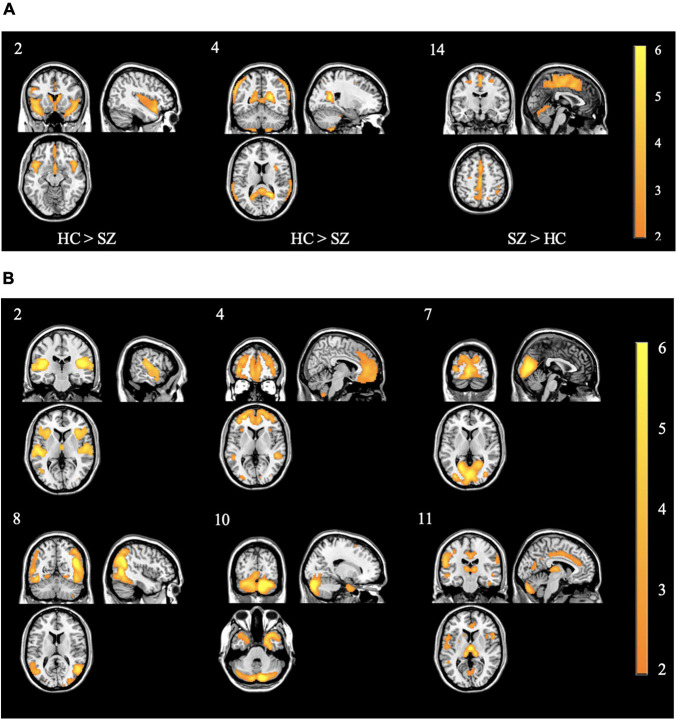
**(A)** Spatial patterns of the concentration components showing control > case effect from the primary SBM decomposition. Component 2 showed the strongest control > case effect Of Note. All images are thresholded at | *z*| > 2. The color bar indicates the color mapping for the component weights. Component numbers are listed in the top left corner of every image. HC: healthy controls; SZ: individuals with schizophrenia. **(B)** Spatial patterns of the volume components showing control > case effect from the primary SBM decomposition. Of note, component 2 showed similar patterns to concentration component 2 in panel **(A)** Of Note. All images are thresholded at | *z*| > 2. The color bar indicates the color mapping for the component weights. Component numbers are listed in the top left corner of every image. All components showed control > case effects.

In the concentration images, three spatial patterns showed a significant effect of diagnosis (see Table 3 and Figure 3A for brain locations). The component with the largest effect (HC > SZ) was located in the superior temporal pole (component 2; *F* = 49.06, *p* < 0.0001). None of the three spatial patterns showed a significant effect of olanzapine equivalents.

**TABLE 3:** Brain labels and peak scores for concentration ICA components showing diagnostic differences.

Component	Diagnostic effect	Peak *x, y, z*	Brain location	Peak | Z|
2	HC > SZ	−43, 16, −17	L superior temporal pole	5.21
	HC > SZ	43, 16, −17	R superior temporal pole	4.51
4	HC > SZ	23, −53, 15	Retrosplenial cortex	6.64
14	SZ > HC	1, −16, 56	SMA	4.52

*ICA, independent component analysis; L, left; R, right; SMA, supplementary motor area; HC, healthy controls; SZ, individuals with schizophrenia.*

In the volume images, six spatial patterns showed a significant effect of diagnosis (see Figure 3B). The components with the largest effects (all HC > SZ) were located in the right prefrontal cortex (*F* = 15.71, *p* = 0.0001), the thalamus (*F* = 11.71, *p* < 0.001), and bilaterally at the superior temporal gyrus (*F* = 6.24, *p* < 0.05). None of the six spatial patterns showed a significant effect of olanzapine equivalents. Refer to Table 4 and Figure 3B for more peak coordinates for the six spatial patterns of diagnostic differences.

**TABLE 4:** Brain labels and peak scores for volume ICA components showing diagnostic differences.

Component	Diagnostic effect	Peak *x, y, z*	Brain location	Peak | Z|
2	HC > SZ	47, −22, 13	R superior temporal gyrus	7.16
	HC > SZ	−42, −28, 8	L superior temporal gyrus	8.17
4	HC > SZ	26, 56, −2	R prefrontal cortex	5.68
	HC > SZ	−26, 56, −3	L prefrontal cortex	6.00
7	HC > SZ	−18, −66, 8	L lingual gyrus	7.83
8	HC > SZ	43, −59, 28	Angular gyrus	9.48
10	HC > SZ	13, −84, −29	Cerebellum/Crus II	9.71
11	HC > SZ	3, −13, 5	R thalamus	7.39
	HC > SZ	3, 28, 23	R anterior cingulum	4.63
	HC > SZ	−57, −18, 23	Somatosensory cortex	4.66

*ICA, independent component analysis; L, left; R, right; HC, healthy controls; SZ, individuals with schizophrenia.*

#### Effect of Quantity

**FIGURE 4:**
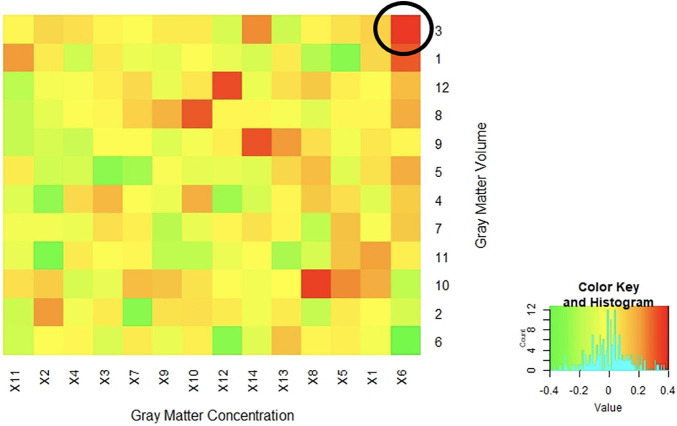
Correlations between Concentration and Volume ICA Components Correlations between the z-weights in the spatial patterns of the gray matter concentration (x axis) and volume (y axis) ICA components from SBM analysis were low to moderate. The highest positive correlation was between spatial patterns in the cerebellum and vermis (*r* = 0.37) circled in the above figure. The majority of the correlations fell between −0.20 < *r* < *0.20* indicating a generally low overlap between the identified regional patterns.

The resulting 168 non-thresholded Pearson’s correlations between the two sets of images ranged from low to moderate with a median correlation of *r* = 0.01 (range from *r* = −0.37 to 0.37). The strongest correlations were with component 6 from the concentration images and component 3 and component 6 from the volume images (*r* = 0.37 and −0.37, respectively). Of note, none of these three (3) components were significant for diagnostic differences, with the majority of the brain region identified being in the cerebellum and vermis (see Figure 4 for more details on correlation results).

### Jaccard Index

**FIGURE 5:**
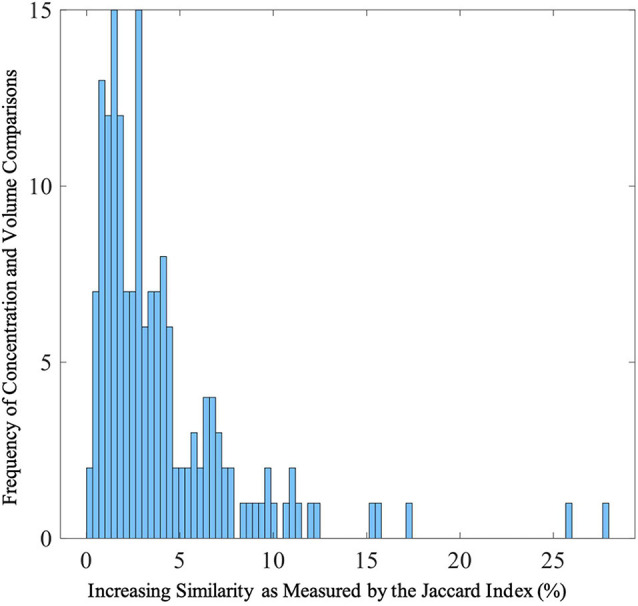
Distribution of Jaccard Indices between Gray Matter Concentration and Volume Spatial Patterns Frequencies of Jaccard Indices showing similarities between gray matter concentration and volume spatial patterns. The range of Jaccard indices was 0.22–27.89% (SD = 4.04%) with a median of 2.73%. The Jaccard indices were calculated from thresholded (*z* > | 2|) voxels that passed threshold.

**FIGURE 6:**
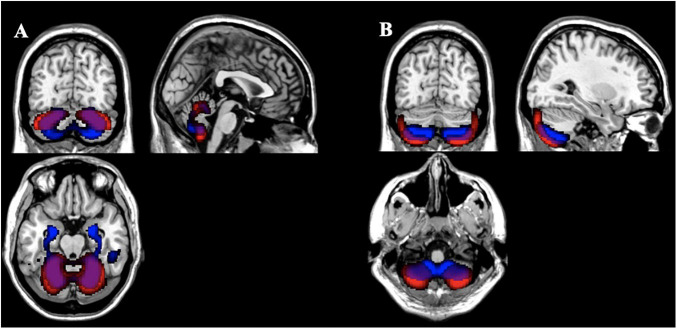
Comparison of Gray Matter Concentration and Volume Spatial Patterns Gray matter concentration (red) and volume (blue) spatial patterns thresholded at *z* > | 2| with the largest Jaccard Index between them [**(A)** 27.89%; **(B)** 25.73%] showing overlap primarily in the cerebellum for those voxels that passed threshold as a union.

After thresholding the spatial patterns at *z* ≥ | 2|, the concentration and volume components showed a median of 2.73% (SD = 4.04%; range: 0.22 to 27.89%) similarity across voxels that passed threshold as a union (see Figure 5 for more details). These results indicate little overlap between the multivariate results. For example, the largest gray matter difference between diagnostic groups (HC > SZ) was found primarily in the gray matter concentration of the superior temporal pole [component 2; *F*(1,123) = 49.39, *p* = 9.42E-11]. However, the Jaccard Index shows minimum similarity (range: 0.74–10.67%) with voxels in this region. The largest overlap (27.89%) between the concentration and volume spatial patterns was primarily in the cerebellum (see Figure 6 for more details).

## Discussion

To date, this is the first study to compare the univariate and multivariate results from gray matter concentration and volume images using a dataset of individuals with schizophrenia. After accounting for age, gender, smoking status, ICV, olanzapine equivalents, and average head motion, the gray matter differences between healthy controls and individuals with schizophrenia were largely seen in the frontal lobe, temporal gyrus, and cerebellum; which is consistent with current schizophrenia literature including previous analyses completed with this dataset (McCarley et al., 1999; Wright et al., 2000; Shenton et al., 2001; Honea et al., 2005; Glahn et al., 2008; Segall et al., 2009; Turner et al., 2012; Gupta et al., 2015). The univariate results showed largely overlapping case/control differences for concentration and volume throughout the cortex (Figure 1). The lack of complete overlap between the images may highlight poor gray and white matter differentiation that may even expand down into the subcortical regions. This finding underlines the fact that volume VBM reflects warping, which is not captured in concentration VBM. However, the voxel by voxel correlations between the quantities had a moderate to high correlation of *r* = 0.56 (range: *r* = 0.00 to 0.99; negative *r* < 4%) indicating sufficient overlap.

Our gray matter concentration VBM and SBM results were largely replications of previous COBRE analysis with small variations in the subcortical regions, brain stem, and insula (Gupta et al., 2015). This lack of exact replication may be explained by the addition of the covariates of ICV, head motion, and smoking status that were not previously explored with this dataset (Gupta et al., 2015). Although there was not exact replication between our concentration spatial patterns and the previous COBRE analyses, the regions identified have been previously recognized as part of larger networks associated with schizophrenia (Wright et al., 2000; Honea et al., 2005; Glahn et al., 2008; Vita et al., 2012). However, the gray matter volume images, primarily from the SBM results, showed marked differences from the previous COBRE analyses and schizophrenia literature.

The multivariate analysis identified very different patterns with diagnostic effects across gray matter concentration and volume. The concentration images identified mostly areas in the superior temporal pole whereas the volume images were mostly in the prefrontal cortex, thalamus, and anterior cingulum. There was moderate overlap in the temporal gyrus and cerebellum (see Figure 6). Overall, the greatest difference between diagnostic groups (HC > SZ) was located in the bilateral superior temporal pole (Component 2 in Table 3). Our analysis showed only one spatial pattern (Component 14 in Table 3) where individuals with schizophrenia had increased gray matter concentration around the motor cortex/sensorimotor area (SMA). This may be explained by noise/movement or alternatively, by an overall reduction of gray matter concentration in the sensorimotor area as individuals age (Douaud et al., 2009). Neither the voxelwise results nor the spatial maps showed an effect of olanzapine equivalents, however, the effect of antipsychotic medication on the cortex is still unclear and in need of further investigation (Roiz-Santiañez et al., 2015).

The addition of Jacobian-scaled modulation can create different gray matter boundaries and therefore, the results may vary depending on how the gray matter is quantified (Good et al., 2001; Radua et al., 2014; Ranlund et al., 2018). Another reason for the differences between gray matter concentration and volume being more pronounced in the multivariate analyses is that, by definition, these analyses are showing patterns of structural changes within the cortex. Therefore, multivariate results indicate divergent neuroanatomical differences. Neural packing varies in density throughout the cortex and can affect these multivariate patterns (Soares and Mann, 1997; Stockmeier and Rajkowska, 2004).

In regards to whole brain automated analysis, another comparison may need to be made between voxel-based morphometry (VBM) and the surface-based analysis of the commonly used FreeSurfer methods^5^. Freesurfer’s segmentation and cortical percolation allows semi-automated regional measures, which have been extensively used in schizophrenia imaging studies (van Erp et al., 2013, 2018). While the atlases included with Freesurfer are useful in producing robust, reproducible effects, the regional averages of thickness or volume do not allow for more specific localization of effects; for example, not all the areas we found in the voxel-wise analysis follow sulcal and gyral boundaries like the caudal anterior cingulate region (see Figure 1 for further details). In the vertex-wise analyses, FreeSurfer is more similar to the voxel-wise analyses in its ability to localize effects, but in contrast to VBM, FreeSurfer provides estimates of localized volume, thickness, and surface area rather than volume and concentration (Fischl et al., 2002; Hutton et al., 2009; Fischl, 2012). There are noted discrepancies between modulated VBM or vertex-wise FS approaches that may be related to surface area, gyrification, and curvature (Palaniyappan and Liddle, 2012; Kong et al., 2015). In addition, the estimate of concentration produces similar results to cortical thickness, but is not reflected in volumetric analyses (Narr et al., 2005).

### Future Directions

Given the results, future studies may seek to examine the lack of overlap in the concentration and volume of gray matter. As previously mentioned, there may be a difference in the neural packing (or potential thinning of gray matter) seen commonly in mood disorders that causes lower gray matter concentration but does not proportionally affect the boundaries of gray matter (Soares and Mann, 1997; Stockmeier and Rajkowska, 2004). Although such differences may not be able to be examined directly in VBM or SBM analyses, post-mortem studies may be able to examine if the differences observed are a result of neural discrepancies. Previous post-mortem studies have identified decreases in the volume and total number of neurons in key areas such as the caudate and putamen in individuals with schizophrenia (Kreczmanski et al., 2007). Neuron density may not be specific to diagnosis and should be examined individually as well as with a variety of populations. Of note, in this study the differences in concentration and volume were not limited to diagnostic differences, indicating that other results (i.e., age differences, gender differences) may also be impacted by how the gray matter is quantified.

With the rise of 7T imaging, a more robust picture regarding the localization of gray matter effects to specific cortical layers (e.g., the gray matter effects in superior temporal gyrus (STG) may not arise from the same mechanism as the effects in the hippocampus, in schizophrenia) may be possible as other studies have shown finer scale organization and more specified delineation of cortical boundaries using 7T imaging (Geyer et al., 2011).

### Limitations

Limitations of this study include examination of differences between gray matter concentration and volume images only in a schizophrenia population. These findings may not generalize to other population groups that do not have as widespread cortical effects as individuals with schizophrenia, or individuals with more severe atrophy. The addition of Jacobian-scaled modulation may not be sensitive enough to detect mesoscopic abnormalities or cortical thinning so these findings may not be generalizable to other imaging studies (Radua et al., 2014). However, these results are still robust enough to warrant replication and should be examined in different populations.

### Conclusion

These results highlight the differences between gray matter concentration and gray matter volume and the importance of distinction between these two quantities in the schizophrenia literature. Furthermore, these results show that there is a relationship between concentration and volume of gray matter but that these two quantities are not interchangeable, especially when examined in multivariate analyses. Therefore, these findings support the recommendation of examining future datasets with both unmodulated and Jacobian-scaled modulated images. Including both quantities could provide a more complete picture of true cortical differences in the populations of interest.

## Data Availability Statement

Publicly available datasets were analyzed in this study. This data can be found here: http://schizconnect.org/.

## Author Contributions

JT and KR-M designed the study. JT and VC acquired the data. KR-M and EZ analyzed the data. KR-M wrote the article, which all authors reviewed. VC consulted on the interpretation. All authors approved the final version to be published and can certify that no other individuals not listed as authors have made substantial contributions to the manuscript.

## Conflict of Interest

The authors declare that the research was conducted in the absence of any commercial or financial relationships that could be construed as a potential conflict of interest.

## Publisher’s Note

All claims expressed in this article are solely those of the authors and do not necessarily represent those of their affiliated organizations, or those of the publisher, the editors and the reviewers. Any product that may be evaluated in this article, or claim that may be made by its manufacturer, is not guaranteed or endorsed by the publisher.

## Footnotes

1
http://coins.trendscenter.org

2
http://schizconnect.org

3
https://www.fil.ion.ucl.ac.uk/spm/software/spm12/

4
http://trendscenter.org/software/gift/

5
http://surfer.nmr.mgh.harvard.edu/
